# An overview of conducting systematic reviews with network meta-analysis

**DOI:** 10.1186/2046-4053-3-109

**Published:** 2014-09-29

**Authors:** Deborah M Caldwell

**Affiliations:** 1 School of Social and Community Medicine, University of Bristol, Canynge Hall, 39 Whately Road, Bristol BS8 2PS, UK

## Introduction

Systematic reviews with network meta-analysis (NMA) are published with increasing frequency in the health care literature. Prior to 2008, very few systematic reviews contained a NMA [1]; however, there has been a marked increase, to mid-2012 Lee recorded 201 published networks [2]. The statistical method has been available since 2002 [3,4] and owes its origins to much earlier work [5,6]. NMA has matured and models are available for all types of underlying data and summary effect measures [7-12] and can be readily implemented in both frequentist and Bayesian frameworks with pre-written programmes available in widely used softwares [8,13-15].

Recently, focus has shifted to making NMA more accessible [16,17]; however, the conduct of systematic reviews for NMA has received less attention [18]. In this special thematic series on network meta-analysis, the editors of *Systematic Reviews* are encouraging submissions of methodological papers concerning the conduct and reporting of meta-analyses and results papers (http://www.systematicreviewsjournal.com/about/update/SysRevCFP). As a preface to the series, this editorial provides an overview of the basic principles of NMA and summarises some of the key challenges for those conducting a systematic review.

### The need for network meta-analysis in comparative effectiveness research

Why has NMA increased in popularity? To illustrate, consider the relative effectiveness of six psychotherapies vs. treatment as usual for treatment of moderate to severe depression [19]. In a pairwise meta-analysis, the systematic reviewer has three synthesis options: (1) “lump” all six psychotherapies together to form a single comparator, (2) conduct six separate pairwise meta-analyses in a single systematic review, or (3) conduct six separate systematic reviews. If the question of interest to the decision-maker is “which psychotherapy should I recommend for depression?” the results of pairwise syntheses do not satisfactorily translate into practice. A clinician does not recommend an “average” psychotherapy to a patient but a specific one, such as cognitive behavioural therapy. To use results from options 2 and 3, the decision-maker must summarise across multiple analyses/reviews without formal assessment of whether the body evidence was coherent or similar enough to form a treatment recommendation. Such an approach makes effect estimates problematic to interpret and is not recommended [20].

NMA came to prominence within this decision-making context [21,22]. NMA is the simultaneous comparison of multiple competing treatments in a single statistical model [23]. In its simplest form, it is the combination of direct and indirect estimates of relative treatment effect, where indirect evidence refers to evidence on treatment C relative to B obtained from A vs. B and A vs. C studies. This is commonly depicted by the equation $\theta_{\mathrm{BC}}^{I}=\theta_{\mathrm{AC}}^{D}-\theta_{\mathrm{AB}}^{D}$ where *θ* denotes the true underlying treatment effect estimate (e.g. log odds ratio, mean difference, etc.) and the superscript either *D*irect or *I*ndirect evidence. If both direct and indirect estimates are available, they can be pooled to produce an internally coherent set of effect estimates of each treatment relative to every other whether or not they have been compared in head-to-head trials. It is also possible to calculate the probability of one treatment being the best for a specific outcome. Treatment options can then be ranked from the best to the worst for each outcome.

### Systematic review process for a network meta-analysis

The rigorous conduct of a standard systematic review should apply equally to a NMA. For example, it is good practice to register a protocol for NMA on a repository such as PROSPERO [24] and report a thorough and reproducible literature search. Inclusion/exclusion criteria for a NMA should also be based on a well-defined population, intervention, comparator, outcome (PICO) research question, since it is the specification of the PICO which ensures the key assumption of transitivity is fulfilled. Transitivity suggests that intervention A is similar when it appears in the A vs. B and A vs. C studies [25]. Transitivity can be examined by comparing the distribution of potential effect modifiers across the different comparisons [26], since if there is an imbalance in the presence of effect modifiers across the A vs. B and A vs. C comparisons, the conclusions about B vs. C may be in doubt. Potential effect modifiers should be pre-specified in a protocol and are usually study level characteristics which are routinely extracted in systematic reviews, such as age, severity, dose, setting, etc. Identifying a lack of transitivity may be difficult and sufficient detail is not always available in published trials to allow a thorough assessment [27,28].

The statistical manifestation of the transitivity assumption is called consistency, which holds when the direct and indirect sources of evidence are in agreement, i.e. ${\hat{\theta }}_{\mathrm{BC}}^{I}={\hat{\theta }}_{\mathrm{AC}}^{D}-{\hat{\theta }}_{\mathrm{AB}}^{D}$ where ^ denotes observed estimates. Transitivity should *always* be examined in NMA; however, it is only possible to assess consistency when there are direct and indirect sources of evidence for a treatment contrast. Thus, inconsistency is a property of “loops” of evidence, here the loop A-B-C [29]. Empirical studies have reported the frequency of statistically significant inconsistency ranging from 2% to 14% of published “loops” of evidence [9,30]. It has been argued, however, that the detection of inconsistency in these studies may reveal less about the reliability of NMA and rather more about the problems associated with systematic review options 2 and 3 identified above [31]. Thus, the assessment of transitivity is of fundamental importance in the conduct of the systematic review.

#### Defining treatments and network size in NMA

Perhaps the biggest deviation from a pairwise systematic review is in the definition of treatments in the network. The identity of each distinct treatment can be preserved in NMA; there is no need to lump across doses or ignore co-treatments in order to conduct analysis. Indeed, the statistical inconsistency noted in empirical studies can often be explained by separating treatments into distinct doses or co-treatments [32].

Treatments included in the network can be divided into a *decision* and *supplementary* set. Treatments within the decision set are the focal treatments of interest to systematic review authors. However, a supplementary set of treatments may also be incorporated into the network to provide additional evidence on relative treatment effects of the decision set. For example, a placebo comparator is rarely of practical clinical interest but its inclusion might (i) connect an otherwise unconnected network of treatments, (ii) increase the precision of the treatment effect estimates of interest if the bulk of the evidence is on placebo comparisons, or (iii) improve estimates of between-trial heterogeneity. Care must be taken to ensure that all treatments in the network are “jointly randomizable” [25]. That is, all treatments should be options for the population considered in the systematic review such that they could reasonably be compared in a single trial.

Sturtz and Bender [33] have referred to network size as an “unsolved issue” in NMA, and it is an area of developing interest [34,35]. The inclusion or exclusion of treatments from the network has the potential to modify treatment effect estimates and the treatment rankings [36]. A meticulous PICO and pre-specified strategy for extending the network [37] will mitigate but not eliminate the risk of *post hoc* inclusion/exclusion of treatments. Where unexpected interventions are identified by the literature search, a sensitivity analysis should be undertaken to examine the impact of its inclusion/exclusion. For the systematic reviewer, the most important consideration in determining network size is likely to be the resource implications of including additional treatments or searching for further evidence to connect existing networks. For example, although a search strategy for decision set treatments is also likely to return those studies also including a supplementary set comparator, the additional resource employed in title screening and eligibility checking is not inconsequential. The larger the network the more intensive the assessment of transitivity, data extraction, risk of bias assessment and tabulation of results is likely to be. Assuming the transitivity assumption holds, the systematic reviewer must balance this extra resource against the benefit of increasing network size.

#### Summarising and reporting network meta-analysis

An important source of guidance for systematic reviewers is the Cochrane Collaborations’ Comparing Multiple Interventions Methods Group. The group focuses on methodology for comparing multiple interventions in Cochrane Intervention Reviews; however, much of the work is generalizable. An example protocol for reviews containing a NMA is available, as is guidance on statistical methods and interpretation and presentation of results (see http://cmimg.cochrane.org/comparing-multiple-interventions-cochrane-reviews). Presenting the results from a systematic review with NMA can be challenging [38,39]. The number of treatments included in NMA can be large; Veroniki’s [9] findings are representative with a range of 4 to 17 treatments (median 6). The number of pairwise comparisons to report from 4 treatments is 6; from 17 treatments, it is 136.

It is commonplace in pairwise systematic reviews to consider the quality of the body of evidence and to summarise the confidence one can place in the conclusions. Attention is turning to how approaches, such as GRADE, can be extended to NMA [40,41]. There are no universally accepted standards for reporting either the methods or results of a NMA, although there are a number of society and national technology assessment organisations who have produced in-house guidance [42,43]. Finally, journal editors and peer reviewers should be mindful that web appendices and supplementary files are a necessity in NMA and they can be large. International initiatives such as the forthcoming extension to PRISMA for reporting of NMA will provide systematic reviewers the much needed guidance here [44].

## Competing interests

DMC is a co-convenor of the Cochrane Collaborations’ Comparing Multiple Interventions Methods Group mentioned in this article.
